# Increased Involvement of *Klebsiella* *pneumoniae* and *Enterococcus* *faecium* in Healthcare-Associated Infections of Intensive Care Units in Taiwan

**DOI:** 10.3390/healthcare9101349

**Published:** 2021-10-11

**Authors:** Yu-Ren Lin, Yen-Yue Lin, Chia-Peng Yu, Ya-Sung Yang, Chun-Gu Cheng, Chun-An Cheng

**Affiliations:** 1 National Defense Medical Center, Department of Emergency Medicine, Taoyuan Armed Forces General Hospital, Taoyuan 32549, Taiwan; alaneli1@yahoo.com.tw (Y.-R.L.); yyline.tw@yahoo.com.tw (Y.-Y.L.); 2 National Defense Medical Center, Department of Emergency Medicine, Tri-Service General Hospital, Taipei 11490, Taiwan; 3 Department of Medical Research, Tri-Service General Hospital, Taipei 11490, Taiwan; yu6641@gmail.com; 4 National Defense Medical Center, School of Public Health, Taipei 11490, Taiwan; 5 National Defense Medical Center, Department of Infection Medicine, Tri-Service General Hospital, Taipei 11490, Taiwan; ysyoung@mail.ndmctsgh.edu.tw; 6 Department of Emergency and Critical Medicine, Wan Fang Hospital, Taipei Medical University, Taipei 11696, Taiwan; 7 National Defense Medical Center, Department of Neurology, Tri-Service General Hospital, Taipei 11490, Taiwan

**Keywords:** healthcare-associated infections, intensive care unit, antimicrobial resistance

## Abstract

Background: Healthcare-associated infections (HAIs) cause increases in length of stay, mortality, and healthcare costs. A previous study conducted in Taiwan obtained similar results to those reported in Korea and Japan in 2015. Changes in microorganisms have been noted in recent years. Understanding the recent condition of HAIs in intensive care units (ICUs) can enable healthcare providers to develop effective infection control protocols to reduce HAIs. Methods: We used the Taiwan Nosocomial Infection Surveillance System to evaluate the incidence densities of HAIs, the proportions of causative pathogens, and the proportions of antimicrobial resistance (AMR). The Poisson regression model was constructed to incidence density, and the chi-square test was used to assess proportion. Results: The incidence density of HAIs decreased 5.7 to 5.4 per 1000 person-days. However, the proportions of *Klebsiella pneumoniae* and *Enterococcus faecium* significantly increased. In addition, the proportions of carbapenem-resistant *K. pneumoniae* and vancomycin-resistant *Enterococcus faecium* significantly increased over time. Conclusion: Analysis of the microorganisms involved in HAIs in ICUs showed elevated proportions of *K. pneumoniae* and *E. faecium* with AMR. Infection control protocols have been implemented for several years and require improvements regarding environmental cleanliness and medical staff prevention.

## 1. Introduction

Healthcare-associated infections (HAIs) generally refer to infections acquired after two days of hospitalization by patients entering the healthcare setting to undergo surgery or other medical treatment. HAIs can also occur after receiving home care or ambulatory care, up to six weeks after discharge, and from 30 days to up to 1 year for device-related infections. In other words, HAIs are infections that patients acquire while they are receiving care for other illnesses [1]. There were 1.7 million HAIs in the United States, and HAIs most commonly include bloodstream infections (BSIs), urinary tract infections (UTIs), and healthcare-acquired pneumonia (HAP), surgical site infections (SSIs), and infections from other parts of the body [2]. *Escherichia coli*, *Staphylococcus aureus*, and *Klebsiella* spp. are the most frequently isolated microorganisms [3]. All these types of infections exhibit various percentages of antimicrobial resistance (AMR), involving pathogens such as extended-spectrum beta-lactamase generating strains of *Enterobacter* spp., *Escherichia coli*, and *K. pneumoniae* [4], methicillin-resistant *S. aureus* (MRSA) [5], carbapenem-resistant *K. pneumoniae* (CRKP), and vancomycin-resistant *Enterococcus faecium* (VREfm) [6], which can complicate treatment [4,5,6]. The inappropriate use of antibiotics causes AMR [7].

HAIs involve infectious opportunistic pathogens, weakened hosts, and implants and prostheses with cross-contamination [8]. The patient’s conditions associated with HAIs include more serious hospitalization in intensive care units (ICUs). The percentage of HAIs in ICUs, approximately 37%, is significantly higher than that in general wards at approximately 5–15% [9]. The most common types of HAIs in ICUs are central line-associated BSIs (CLABSIs), catheter-associated UTIs (CAUTIs), and ventilation-associated pneumonia (VAP). HAIs increase length of stay, cost, and mortality. Additionally, they increase patients’ disease severity [10].

Infection control monitoring and prevention procedures are important in preventing HAIs. In addition, the identification of resistant pathogens in HAIs is essential. The AMR of HAIs can be studied to understand the local epidemiology and improve local infection control practices (ICPs). Current control and prevention practices include antimicrobial use, ICPs, environmental cleaning, and efforts to reduce community burden. Trends in the incidence of HAIs, device-induced HAIs (DIHAIs), and AMR have been objectively described and compared with those in other countries [11].

A recent study on HAIs in ICUs from the Taiwan Nosocomial Infection Surveillance System (TNIS) found similar percentages to those reported in Korea and Japan in 2015 [12]. An understanding of recent conditions regarding different microorganisms and their AMR could provide the government with a disease control policy. We investigated the trends in HAIs in ICUs since 2015 to understand the changes in Taiwan.

We aimed to identify recent pathogens of HAIs and AMR in the ICUs of medical centers and regional hospitals in Taiwan. We investigated the trends in HAIs, DIHAIs, and AMR to reveal the changes in HAIs. The prevention of HAIs is a priority; the incidences of HAIs involving different microorganisms and the proportions of AMR need to be recognized to adopt ICPs for HAI reduction in ICUs.

## 2. Materials and Methods

The TNIS was established in 2007 to monitor the occurrence of HAIs, and we examined publicly available annual summary data on HAIs reported by medical centers and regional hospitals in Taiwan [13]. However, the data did not include HAI data from local hospitals. We used demographic data on HAIs and patient-specific cultures and antimicrobial susceptibility results from reporting hospitals in the TNIS from 2015 to 2019.

We included the following HAIs: BSIs, UTIs, HAP, SSIs, and infections at other sites. We collected data on the overall incidence, causative pathogens, and AMR of HAIs in the ICUs of medical centers and regional hospitals. Information was obtained from 104 hospitals in this study, accounting for over 21.5% of Taiwan’s hospitals.

This study did not require ethical approval because it was based on information that was freely available in the public domain and involved the analysis of open-source datasets, in which the data have been properly anonymized.

### 2.1. Definitions

The incidence density of HAIs was calculated as the overall number of HAI episodes divided by overall number of hospitalization patient-days (persons/1000 patient-days). The proportion of microorganisms was the number of individual microorganisms divided by the overall number of microorganisms every year. The incidence density of DIHAIs was calculated as the overall number of DIHAI episodes divided by the overall number of hospitalization DIHAIs patient-days. The device use rate was calculated as the number of device-used person-days divided by the number of hospitalization person-days. The proportion of antimicrobial-resistant microorganisms of HAIs was calculated as the number of AMR microorganisms divided by the total number of individual microorganisms every year.

### 2.2. Statistical Analyses

The Poisson regression model with log-linear regression for count data was used to assess trends in HAI and DIHAI incidence densities. The distributions of pathogens and their AMR over time were determined. Differences between years in the proportions of microorganisms and AMR microorganisms were examined using the Mantel–Haenszel chi-square for linear trends. The significant level was set at *p* < 0.05. The analysis was conducted using IBM SPSS version 22. (Asia Analytics Taiwan Ltd., Taipei, Taiwan).

## 3. Results

**Figure 1:**
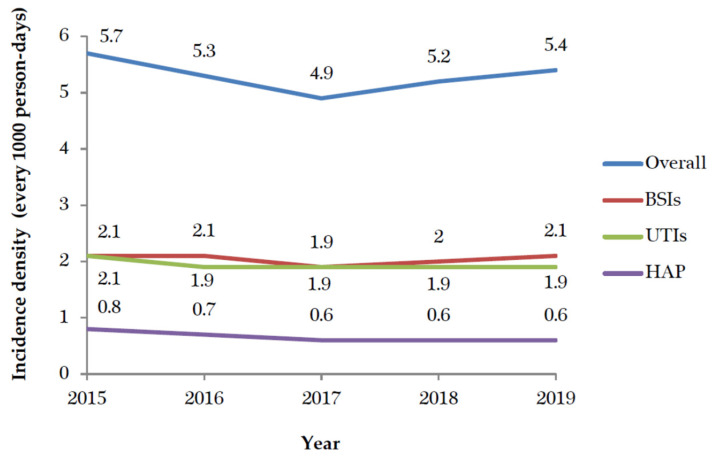
The trends of healthcare-associated infections from 2015 to 2019. BSIs: bloodstream infections; UTIs: urinary tract infections; HAP: hospital-acquired pneumonia.

**Figure 2:**
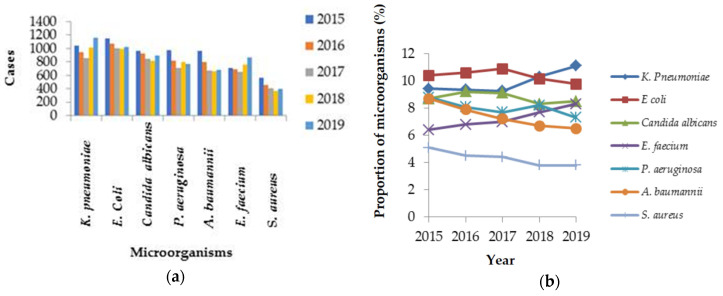
The numbers (**a**) and proportions (**b**) of different microorganisms in healthcare-associated infections from 2015 to 2019.

The incidence density of HAIs in Taiwanese ICUs significantly decreased from 5.7% to 5.4% from 2015 to 2019 (*p* < 0.001). No significant change was found in the incidence density of BSI (*p* = 0.134). The incidence densities of UTIs and HAP significantly decreased over time (*p* < 0.001) (Figure 1). In the ICUs of medical centers, the most common HAIs were BSIs (44.4%) and UTIs (32.9%). In regional hospitals, UTIs (39.5%) and BSIs (36.3%) were predominant. The numbers of episodes involving *K. pneumoniae* (1045 to 1156) and *E. faecium* (711 to 867) significantly increased between 2015 and 2019, whereas the numbers of episodes involving *Staphylococcus aureus*, *Pseudomonas aeruginosa*, and *Acinetobacter baumannii* significantly decreased. The incidence densities and percentages of DIHAIs in ICUs significantly decreased over time (Table 1). The percentages of *K. pneumoniae* (9.4% to 11.1%) and *E. faecium* (6.4% to 8.3%) significantly increased over time. In contrast, the proportions of HAIs involving *Pseudomonas aeruginosa*, *Acinetobacter baumannii*, and *Staphylococcus aureus* significantly decreased. The proportion of *Candida albicans* and *E. coli* in HAIs of ICUs decreased, but the decreases were insignificant (Figure 2).

In 2019, the incidence densities of HAIs were higher than the average incidence density of HAIs (5.4‰) in the medical ICU (MICU) (5.9‰) (*p* < 0.001), and surgical ICU (SICU) (6.5‰) (*p* < 0.001). The incidence densities of HAIs in the pediatric ICU (PICU) and SICU were significantly decreased compared with 2015, decreasing from 2.6‰ to 2.1‰ (*p* < 0.001) and from 6.9‰ to 6.5‰ (*p* = 0.015), respectively. The incidence density of CLABSIs was higher than the average (3.18 ‰) of CLABSIs in the MICU (3.7‰) (*p* < 0.001). Furthermore, the incidence density of CAUTIs in MICU (3.1‰) (*p* = 0.001) was higher than the average (2.73‰). The incidence densities of VAP in the SICU (0.85‰) (*p* = 0.018) and GICU (0.98‰) (*p* < 0.001) were higher than the average (0.7‰) (Table 2).

The relative percentages of microorganisms of HAIs were compared between 2015 and 2019. *K. pneumoniae* was the most common pathogen of bloodstream HAIs in ICUs, increasing from 9.6% to 11.9% of the total, with an absolute 2.3% increase. *A. baumanni* was the second most common pathogen in bloodstream HAIs in ICUs and underwent an absolute 1.9%, decreasing from 10.4% to 8.5%. The third most common microorganism in bloodstream HAIs in ICUs was *E. faecium*, which increased from 7.2% to 8.5%, an absolute 1.3% increase. Candida otherwise specified was the fourth most common pathogen in bloodstream HAIs in ICUs, it increased from 5.9% to 7.9% over time, with an absolute 2% increase. *E. coli* was the fifth most common microorganism among bloodstream HAIs.

*E. coli* was the most common microorganism in urinary tract HAIs in ICUs and underwent an absolute 1.7% reduction over time, decreasing from 19.8% to 18.1%. The second common pathogen in ICU urinary tract HAIs was *C. albicans*. *E. faecium* was the third most common pathogen in these HAIs and increased from 8.5% to 10.1%, with an absolute 1.6% increase. *K. pneumoniae* was the fourth most common pathogen in urinary tract HAIs in ICUs; it increased from 7.3% to 9.6%, with an absolute 2.3% increase. The fifth most common pathogen in ICU urinary tract HAIs was Candida otherwise specified; it increased from 7.2% to 9.3%, with an absolute 2.1% increase.

*P. aeruginosa* was the most common pathogen in HAP in ICUs, followed by *K. pneumoniae*. *A. baumannii* was the third most common microorganism in HAP infection in ICUs, accounting for 18% to 14.2% of cases, with an absolute 3.8% reduction. *S. aureus* and Enterobacter species were the fourth and fifth most common HAP pathogens in ICUs. Among the SSIs in ICUs, *P. aeruginosa* was the most common pathogen, followed by *K. pneumoniae*, *E. coli*, and *E. faecium* were the third most common pathogens in ICU SSIs. *E. faecium* was increased from 4.2% to 8.4%, with an absolute 4.2% increase. *A. baumannii* was the fifth most common pathogen in ICU SSIs.

*K. pneumoniae* ranked second to first in BSIs, third to second in the HAP, and fifth to fourth in the UTIs from 2015 to 2019; *E. faecium* ranked in third position in BSIs, UTIs, and SSIs in 2019 (Table 3).

**Figure 3:**
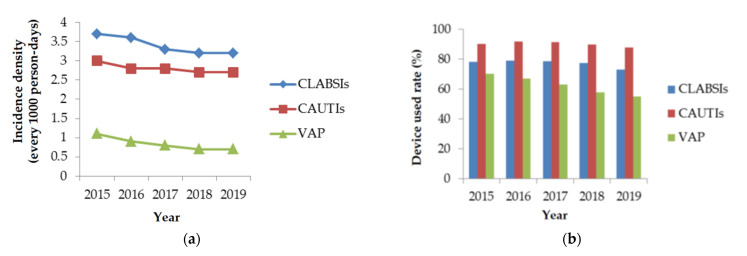
The trend (**a**) and device use rate (**b**) of device-induced healthcare-associated infections from 2015 to 2019. CLABSI: central line-associated bloodstream infections; CAUTIs: catheter-associated urinary tract infections; VAP: ventilator-associated pneumonia.

**Figure 4:**
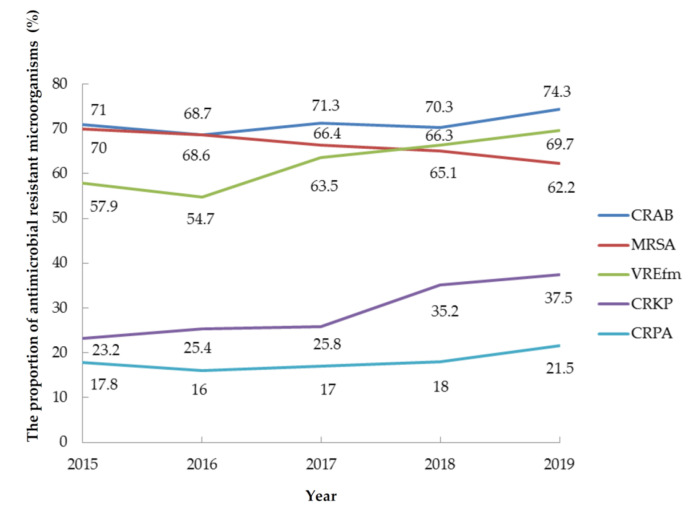
The proportions of antimicrobial-resistant microorganisms in hospital-associated infections from 2015 to 2019. CRAB: carbapenem-resistant *A. baumannii*; MRSA: methicillin-resistant *S. aureus*; VREfm: vancomycin-resistant *E. faecium*; CRKP: carbapenem-resistant *Klebsiella pneumoniae*; CRPA: carbapenem-resistant *P. aeruginosa*.

The incidence densities of DIHAIs showed decreasing trends from 2015 to 2019 (*p* < 0.001) (Figure 3). The percentage of central line use rate in CLABSIs was reduced from 78.3% to 73.1%. The percentage of urinary catheter use rate in CAUTIs was reduced from 90.3% to 87.9%. The percentage of ventilation use rate in VAP was reduced from 70.2% to 54.9% from 2015 to 2019 (*p* < 0.001). There were significant increases in the number of isolates of CRKP and vancomycin-resistant *Enterococcus* (VRE) from 2015 to 2019. The percentage of CRKP isolates increased from 23.2% to 37.5%, representing 61.6% (absolute 14.3%) (*p* < 0.001). The percentage of VRE isolates increased from 35.7% to 47.6%, representing a 33.3% increase (absolute 11.9%), and that of VREfm isolates increased from 57.9% to 69.7%, representing a 20.4% increase (absolute 11.8%) (*p* < 0.001). The percentage of carbapenem-resistant *E. coli* isolates increased by 90.5% from 2.1% to 4%, corresponding to an absolute increase of 1.9% (*p* = 0.008). The percentage of MRSA isolates decreased from 70% to 62.2%, representing an 11.1% decrease (absolute 7.8%); this decreasing trend was insignificant (*p* = 0.107). The percentage of carbapenem-resistant *P*. *aeruginosa* isolates increased from 17.8% to 21.5%, representing an insignificant increase of 20.8% (absolute 3.7%) (*p* = 0.055). The percentage of carbapenem-resistant *A. baumanni* isolates increased from 71% to 74.3%, representing an insignificant increase of 4.6% (absolute 3.3%) (*p* = 0.147) (Figure 4).

## 4. Discussion

The total proportion of *K. pneumoniae* and *E. faecium* in HAIs in ICUs increased. The changes were driven by an increase in *K. pneumoniae* and *E. faecium* in HAIs representing BSIs, UTIs, and SSIs. The AMR of CRKP and VREfm significantly increased, which may have contributed to these trends. Although ICPs and antimicrobial stewardship have been implemented for years [7], improvements and aggressive procedures for HAIs in Taiwanese ICUs remain needed.

The TNIS can be used to evaluate the epidemiologic trends of HAIs and internationally comparable surveillance indicators. The most frequent type of infection in hospitals in the United States are UTIs (36%), followed by SSIs (20%) and BSIs and pneumonia (both 11%) [8]. BSIs rather than UTIs were the HAIs with the highest frequency in Taiwan, in contrast to the pattern in the USA. In Iranian ICUs, the incidence of BSIs, UTIs, and HAP has been reported to be 2.21, 3.32, and 2.55, respectively, every 1000 person-days [14]. The incidence densities of BSIs, UTIs, and HAP in the ICUs of Taiwan were 2.1, 1.9, and 0.6, respectively, every 1000 person-days. The HAIs in Taiwan have a lower incidence density than those in Iran. In addition, the values for the MICU and general ICU in Taiwan are higher than those in Iran [14]. The most common HAIs of Taiwanese ICUs occurred in the SICU, followed by the MICU. Although the incidence densities have decreased over time in the SICU and PICU; however, aggressive ICPs and monitoring are needed in the SICU.

*E. coli* (18%), *S. aureus* (12%), and *Klebsiella* spp (9%) were the most frequent HAI pathogens reported in Atlanta of the United States [3]. In Ukraine, *K. pneumoniae* was the most common pathogen reported, accounting for 21.8% of HAIs, followed by *A. baumannii* (14.3%), *P. aeruginosa* (12.4%), and *E. coli* (9.4%) [15]. The most common causative bacteria identified in Thailand were Gram-negative bacteria, of which *K. pneumoniae* (18.5%) was the most common, followed by *A. baumannii* (17.8%) and *P. aeruginosa* (12.6%) [16]. Our study found that *K. pneumoniae* was the most common microorganism of HAIs in Taiwan, similar to results in Ukraine and Thailand, but different from those in the United States and Europe, where *P. aeruginosa* has been identified as the most common microorganism [17].

In a 2007 study in Europe, the most frequently isolated microorganisms were *P. aeruginosa* in ICU-HAP episodes, coagulase-negative staphylococci in ICU-BSIs, and *E. coli* in ICU-UTIs [18]. These findings are similar to the results for HAP and UTIs in our study. In our study, the most common microorganism of BSIs in ICUs was *K. pneumoniae*, different from the pattern in Europe. The percentage of BSIs involving *E. faecium* was found to be 5.4% in 2010 in the USA [19]. Our study revealed a higher prevalence (8.5%). The percentage of UTIs due to *E. faecium* was 39.9% in a 2014 study in Australia [20]. Our study identified a lower prevalence (10.1%).

The occurrence of HAIs is associated with invasive procedures, and monitoring for CLABSI, CAUTI, and VAP is needed. Infectious disease control requires appropriate ventilation devices, catheters, central lines, timely catheter removal, regular education programs following infection control guilds, monitoring of water and diet quality, and focused care of patients with AMR infections [21]. Our study revealed that DIHAIs were significantly reduced after ICP promotion in recent years in Taiwan.

The proportions of *K. pneumoniae* and *E. faecium* among HAIs increased from 2015 to 2019, whereas those of *P. aeruginosa*, *A. baumannii*, and *S. aureus* decreased. In addition, the proportion of CRKP and VREfm significantly increased. The proportions of CRPA, CRAB, and MRSA were insignificantly decreased. Although CR *E. coli* significantly increased, it increased only to 4%; this percentage is lower than the percentage of *E. coli* related HAIs, which changed from 10.4% to 9.8% (*p* = 0.098).

Carbapenem antibiotics are used to treat extended-spectrum β-lactamase bacterial infections. The mortality of CRKP infection is higher, with a value of 27.3% reported in a previous study [22]. Compared with carbapenem-susceptible *K. pneumoniae*, CRKP in BSI is associated with significantly worse 2-week survival [23]. Difficult-to-treat Gram-negative BSI and more severe lower respiration tract infection are associated with increased in-hospital mortality within one month and appropriate empirical antibiotics related to lower mortality [24]. In Europe, carbapenem resistance was reported in 15% of *Klebsiella* spp. isolates, 26% of *P. aeruginosa* isolates, and 64% of *A. baumannii* isolates. In Ukraine, 29.3% of isolates were found to be CRKP [15], and the percentage of CR Enterobacter isolates in a USA study was 26.7% [25]. Our study revealed that carbapenem resistance among *A. baumannii* (74.3%) and *K.* *pneuminae* (37.5%) was higher in 2019 than in 2015. KPC-2 and OXA-48 have been the most common genes associated with resistance over the last 20 years in Taiwan [26]. The risk factors for CRKP include ICU hospitalization, device use, and antibiotics use [27]. Ceftazidime/avibactam can treat CRKP. The incidence of newly acquired VRE during ICU stay in Taiwan University Hospital was 21.9 per 1000 patient-days in 2009 [28]. The proportion of VRE in Taiwan is 47.6% higher than that (17.3%) in Saudi Arabia [29] and 33.6% in the USA [25]. MLST 414 is the most predominant VRE strain. The increased VRE prevalence is due to cross-transmission of VRE clones ST 414, 78, and 18 by undetected VRE carriers. *E. faecium* caused 49.1% of enterococcal BSIs in a study in Australia [20]. A previous study in Taiwan found that VREfm increased from 0.3% in 2004 to 24.9% in 2010 [30]. The frequency of VREfm was 75.6% in the United States during 2014 [31], 19.0% in Europe in 2018 [32], and 59.1% in Switzerland with frequent tourism [33]. The risk factors for VREfm in Germany, with a frequency of 26.1% in 2017, were found to be the hospitalization in southwest and southeast areas, age 40–59 years vs. lower, specialist care, and prevention and rehabilitation care [34]. Our study found that the proportion of VREfm was similar to the proportion in the USA, but higher than that in Europe. The most common area of CRKP (52.2%) and VREfm (79.4%) was North Taiwan; the reason might be the higher proportion of medical centers in North Taiwan than in other regions. Regarding the onset of VREfm BSI in the ICU, a 30-day mortality rate of 13.2% has been reported, with an odds ratio of 4.2 (95% confidence interval 1.7–10) [35]. CRKP and VRE are threats to public health. The increasing trends of CRKP and VRE may reflect the extensive use of carbapenem and vancomycin. Effective monitoring, feedback regarding antimicrobial stewardship programs, and aggressive ICPs are needed to reduce CRKP and VRE.

The most frequent route of transmission of HAIs is direct contact. *K.* *pneumoniae* and *E.* *faecium* can form biofilms with adaptations to hospital conditions that serve as vehicles of transmission and dissemination in the hospital setting. *K. pneumoniae* can remain on a surface for two hours to two and a half years, and *E. faecium* can persist on a surface for five days to four months [36]. Cross-transmission of infections causes the contamination of equipment, bed linens, and air droplets, and infections are spread by medical staff and visitors. VRE is the most common multiresistant microorganism isolated in contaminated rooms [37]. It is essential to reduce infection carriage and spread. A clean environment is important in HAIs prevention. The use of nonflammable alcohol vapor in carbon dioxide, H_2_O_2_, and ultraviolet C for surface decontamination has been shown in previous studies to reduce environmental microorganisms [38,39,40,41,42]. Environmental improvement, screening for CRKP and VREfm carriage, interinstitutional infection control measures, universal health education, and suitable antimicrobial agent use can lead to reduced harm from infectious diseases.

Three aspects of infection are the ease with which an agent can infect a host with dysfunctional immunity, the infectious agent, and the infection routes. Clean care is safer care. It is important to implement hand washing to maintain hand hygiene. Every healthcare staff member needs to wear facial and oral masks, protective clothing, and gloves. The risk factors for HAIs include age older than 65 years, hospitalization from the emergency room, ICU admission with higher risk, induced devices, surgical procedure, immunocompromised status, and underlying diseases. One-third of HAIs are considered preventable. Hand cleaning, appropriate antibiotic use, patient isolations, the use of appropriate personal protective equipment, and environmental cleaning with disinfection procedures are performed by personnel for infection control [43].

This study used national surveillance data to evaluate the trends of HAIs of ICUs in Taiwan, and the results support recent data from elsewhere in Asia. There are some limitations to this study. First, it included data only from regional hospitals and medical centers, representing approximately 21.5% of all hospitals in Taiwan, and data from local hospitals need to be accessed in the future. However, only 13.6% of all ICU hospitalizations resulted in patients admitted to the ICU at local hospitals in Taiwan [44]. The HAIs in this study cover 86% of ICU admissions to medical centers and regional hospitals and seem representative of Taiwan. Second, data on the patients’ clinical conditions and antibiotic effects were not available; the past study found that appropriate antibiotic therapy did not affect mortality due to CRKP bacteriuria in ICUs, yielding controversial conclusions [45]. A registered study could address this limitation. Third, the proposed association between AMR and microorganisms is speculative, and future studies are needed to confirm the hypothesis.

## 5. Conclusions

This study found that *K. pneumoniae* and *E. faecium* in HAIs exhibited significant increases over a five-year period. The AMR of CRKP and VRE is the most important factor influencing cross-transmission. During the COVID-19 outbreak in March 2020, the prevention of HAIs and subsequent complications due to infection was essential for patient survival. ICPs need to be implemented in accordance with recent findings. Target preventative measures with clean environments and the appropriate use of antibiotics to reduce AMR need to be implemented. It is important to understand the latest condition of HAIs in different countries. These present findings can provide other countries with alerts to these conditions and allow them to develop strategies to prevent HAIs in ICUs.

## Figures and Tables

**Table 1:** Healthcare-associated infections were compared between 2015 and 2019.

	2019	2015	*p*
Incidence density of healthcare-associated infections (‰)	5.4	5.7	<0.001 *
Incidence density of bloodstream infections (‰)	2.1	2.1	0.728
Incidence density of hospital-associated pneumonia (‰)	0.6	0.8	<0.001 *
Incidence density of urinary tract infections (‰)	1.9	2.1	0.001 *
Incidence density of central line-associated bloodstream infections (‰)	3.2	3.7	<0.001 *
Incidence density of ventilator-associated pneumonia (‰)	0.7	1.1	<0.001 *
Incidence density of catheter-associated urinary tract infections (‰)	2.7	3	<0.001 *
Percentage of device use rate in central line-associated bloodstream infections	73.1	78.3	<0.001 *
Percentage of device use rate in ventilator-associated pneumonia	54.9	70.2	<0.001 *
Percentage of device use rate in catheter-associated urinary tract infections	87.9	90.3	<0.001 *
*Klebsiella pneumoniae*	1156	1045	<0.001 *
*Escherichia coli*	1021	1152	0.118
*Candida albicans*	894	963	0.695
*Enterococcus faecium*	867	711	<0.001 *
*Pseudomonas aeruginosa*	766	976	<0.001 *
*Acinetobacter baumanni* *i*	684	963	<0.001 *
*Staphylococcus aureus*	402	563	<0.001 *
CRKP	37.5%	23.2%	<0.001 *
CRAB	74.3%	71%	0.147
CRPA	21.5%	17.8%	0.038
CR *E. coli*	4%	2.1%	0.008 *
MRSA	62.2%	70%	<0.001 *
Vancomycin-resistant *Enterococcus*	47.6%	35.7%	<0.001 *
Vancomycin-resistant *Enterococcus faecium*	69.7%	57.9%	<0.001 *

CRKP: carbapenem-resistant *Klebsiella pneumoniae*; CRAB: carbapenem-resistant *Acinetobacter baumannii*; CRPA: carbapenem-resistant *Pseudomonas aeruginosa*; CR *E. coli*: carbapenem-resistant *Escherichia coli*; MRSA: methicillin-resistant *Staphylococcus aureus*. * *p* < 0.05.

**Table 2:** The incidence densities of device-induced healthcare-associated infections in different types of intensive care units during 2019.

	CLABSIs (per 1000 Person-Days)	CAUTIs (per 1000 Person-Days)	VAP (per 1000 Person-Days)
Medical intensive care units	3.7	3.1	0.41
Surgical intensive care units	3.16	2.54	0.85
Cardiac intensive care units	3.21	2.98	0.29
Pediatric intensive care units	1.99	1.45	0.58
General intensive care units	3.02	2.59	0.98
Average	3.18	2.73	0.7

CLABSIs: central line-associated bloodstream infections; CAUTIs: catheter-associated urinary tract infections; VAP: ventilation-associated pneumonia.

**Table 3:** The distributions of the top five causative pathogens and sites of infection in 2015 and 2019.

2019	Organism		2015	Organism	
Urinary tract infections	Total (3744)	Percentage		Total (3990)	Percentage
1	*Escherichia coli*	18.1	1	*Escherichia coli*	19.8
2	*Candida albicans*	15	2	*Candida albicans*	16.9
3	*Enterococcus faecium*	10.1	3	*Enterococcus faecium*	8.5
4	*Klebsiella pneumoniae*	9.6	4	*Pseudomonas aeruginosa*	7.4
5	Candida otherwise specified	9.3	5	*Klebsiella pneumoniae*	7.3
Bloodstream infections	Total (4272)			Total (4138)	
1	*Klebsiella pneumoniae*	11.9	1	*Acinetobacter baumanni* *i*	10.4
2	*Acinetobacter baumanni* *i*	8.5	2	*Klebsiella pneumoniae*	9.6
3	*Enterococcus faecium*	8.5	3	*Enterococcus faecium*	7.2
4	Candida otherwise specified	7.9	4	*Staphylococcus aureus*	6.5
5	*Escherichia coli*	5.2	5	*Candida albicans*	6.2
Healthcare-acquired pneumonia	Toral (929)			Toral (1397)	
1	*Pseudomonas aeruginosa*	19.9	1	*Pseudomonas aeruginosa*	22.5
2	*Klebsiella pneumoniae*	17.5	2	*Acinetobacter baumanni* *i*	18
3	*Acinetobacter baumanni* *i*	14.2	3	*Klebsiella pneumoniae*	16.2
4	*Staphylococcus aureus*	8.2	4	*Staphylococcus aureus*	9
5	Enterobacter species	5.9	5	Enterobacter species	6.2
Surgical site infections	Total (699)			Total (698)	
1	*Pseudomonas aeruginosa*	11.7	1	*Pseudomonas aeruginosa*	12.8
2	*Klebsiella pneumoniae*	10.3	2	*Klebsiella pneumoniae*	10.2
3	*Enterococcus faecium*	8.4	3	*Escherichia coli*	9.6
3	*Escherichia coli*	8.4	4	*E* *nterobacter cloacae*	8.2
5	*Acinetobacter baumanni* *i*	6.3	5	*Staphylococcus aureus*	7.2

## Data Availability

Annul reports of Taiwan Nosocomial Infections Surveillance System: https://www.cdc.gov.tw/Category/MPage/4G8HuDdUN1k4xaBJhbPzKQ (accessed on 1 June 2021).
